# Whole-Body Cryostimulation Improves Inflammatory Endothelium Parameters and Decreases Oxidative Stress in Healthy Subjects

**DOI:** 10.3390/antiox9121308

**Published:** 2020-12-21

**Authors:** Agata Stanek, Tomasz Wielkoszyński, Stanisław Bartuś, Armand Cholewka

**Affiliations:** 1 Department and Clinic of Internal Medicine, Angiology and Physical Medicine, Faculty of Medical Sciences in Zabrze, Medical University of Silesia, Batorego 15 Street, 41-902 Bytom, Poland; 2 Higher School of Strategic Planning in Dąbrowa Górnicza, Kościelna 6 Street, 41-300 Dąbrowa Górnicza, Poland; t.wielkoszynski@wielkoszynski.pl; 3 Second Department of Cardiology, Institute of Cardiology, Jagiellonian University Medical College, Kopernika 17 Street, 31-501 Krakow, Poland; stanislaw.bartus@uj.edu.pl; 4 Faculty of Science and Technology, University of Silesia, Bankowa 12 Street, 40-007 Katowice, Poland; armand.cholewka@gmail.com

**Keywords:** whole-body cryostimulation, wellness, endothelium parameters, oxidative stress

## Abstract

Background: The purpose of this study was to estimate the effect of whole-body cryostimulation (WBC) and subsequent kinesiotherapy on inflammatory endothelium and oxidative stress parameters in healthy subjects. Methods: The effects of ten WBC procedures lasting 3 min per day and followed by a 60-min session of kinesiotherapy on oxidative stress and inflammatory endothelium parameters in healthy subjects (WBC group *n* = 32) were analyzed. The WBC group was compared to a kinesiotherapy only (KT; *n* = 16) group. The following parameters were estimated one day before the start, and one day after the completion of the studies: oxidative stress parameters (the total antioxidant capacity of plasma (FRAP), paraoxonase-1 activity (PON-1), and total oxidative status (TOS)) and inflammatory endothelium parameters (myeloperoxidase activity (MPO), serum amyloid A (SAA), and sCD40L levels). Results: A significant decrease of PON-1 and MPO activities and TOS, SAA, and sCD40L levels as well as a significant FRAP increase were observed in the WBC group after the treatment. In addition, the SAA levels and PON-1 activity decreased significantly after the treatment in both groups, but the observed decrease of these parameters in the WBC group was higher in comparison to the KT group. Conclusion: WBC procedures have a beneficial impact on inflammatory endothelium and oxidative stress parameters in healthy subjects, therefore they may be used as a wellness method.

## 1. Introduction

The history of using of cold in medicine goes back to ancient times in Egypt where it was used from 2500 B.C. It was at this time when cold was connected with anti-inflammatory and pain-relieving effects at injury sites. A few hundred years later, in the 5th century B.C., Hippocrates used cold to decrease oedema, bleeding, and pain. At that time, cold remedies such as snow, ice-water mixtures, and cold water were used. D. J. Lorrey, the Napoleonic surgeon during the Russian campaign also noticed that covering the limbs of wounded soldiers with ice or snow reduced bleeding and pain during their amputation [1].

The development of modern cryostimulation started at the end of the 19th century when physicists discovered how to condense gases. A huge contribution to this was made by Karol Olszewski and Zygmunt Wroblewski (among other scientists), who in 1883 condensed oxygen and nitrogen. Since that time industrial-scale production and the ability to store the gases has become possible, allowing for practical uses of the gases, such as in medicine. Later, in 1907, Whitehouse constructed the first device which allowed for the release of liquid vapors. This was used to treat superficially-located neoplasms and some dermatological diseases [1].

The beginnings of whole-body cryotherapy (WBC) go back to 1978 when T. Yamauchi used, for the first time, a cryochamber to treat patients with rheumatoid arthritis. Four years later R. Fricke introduced this method in Germany. In Poland, the first cryochamber was constructed in 1989. At that time, it was the second cryochamber in Europe and the third one in the world [1,2]. WBC exposes the total human body to extreme cold (usually −120 to −160 °C) for a short time in a special cryochamber to induce physiological and psychological benefits. During WBC procedures the more receptors that are in the overcooling zone, the better the result of treatment. Recently, the term “cryostimulation” has appeared alongside the term “cryotherapy”. It should be explained that cold-stimulation mechanisms are the same in both cases, but the term “cryostimulation” is targeted toward healthy subjects, whereas cryotherapy involves patients [3].

So far, WBC procedures have been commonly used in the treatment of rheumatic and inflammatory diseases such as rheumatoid arthritis [4], fibromyalgia [5,6], and ankylosing spondylitis [7,8] as well as multiple sclerosis [9]. The method has also been used in psychiatry to improve mental well-being [10] and memory and to decrease levels of fear, anxiety, and excitability as well as to increase perception [11]. WBC procedures are also used increasingly often in sports medicine and are applied in the treatment of both acute and chronic soft tissue injuries. However, the mechanism of the beneficial impact of cryogenic temperature on muscle injuries caused by training overload is not yet known in detail. WBC procedures have a vast scope of application; apart from post-traumatic rehabilitation of injured locomotor system, they are also in the training of professional athletes. A significant factor is a decrease in the intensity of post-traumatic pain and edema, to which athletes are especially exposed during their training, as well as a subjective improvement in general condition and better exercise tolerance [2,12,13,14].

According to present knowledge, the absolute contraindications for applying WBC are cold intolerance, cryoglobulinemia, cryofibrynogemia, venous thromboembolism, claustrophobia, acute diseases of respiratory system, active tuberculosis, hypothyroidism, local disorders of blood supply, wounds, neoplastic diseases, cachexy, hypothermia, severe anemia, and mental diseases restricting communication with the subject, as well as diseases of the cardiovascular system (moderate arterial hypertension, unstable angina, circulatory failure, and cardiac rhythm disorders such as sinus tachycardia of over 100 beats per minute) [1,2,3].

At the moment, WBC is being used to maintain well-being in healthy subjects [1,15]. There are, however, only a few studies partially describing the mechanisms of WBC action but the whole process is not yet fully known.

So far, it has been proved that WBC procedures may have a beneficial influence on oxidative stress [15,16], inflammatory processes [7,17], lipid profile [18,19], quality of sleep [20], and the endocrine system [21,22] as well as skin [23] in healthy subjects. WBC procedures may also result in regression to tiredness and reduced willingness to participate in physical activity and exercise as well as readiness to co-operate with a doctor or physiotherapist during the rehabilitation process [1].

Taking the above findings into consideration, the purpose of the present study was to analyze the impact of WBC procedures with a subsequent session of kinesiotherapy, on inflammatory endothelium function as well as on some chosen oxidative stress parameters in healthy subjects.

## 2. Materials and Methods

### 2.1. Subjects

The research protocol was performed in compliance with the Declaration of Helsinki and was approved by the Bioethical Committee of the Medical University of Silesia in Katowice (permissions no. NN-013-149/I/02 and NN-6501-93/I/07, Poland). All subjects gave written consent for inclusion in the research. 

Forty-eight healthy non-smoking male subjects were included in the research. They were divided randomly by a physician into two groups with an allocation ratio of 2:1. The first group consisted of 32 healthy subjects (WBC group, mean age 43 ± 3.9 years) who underwent whole-body cryostimulation procedures with subsequent kinesiotherapy. The second group consisted of 16 healthy subjects who were only exposed to kinesiotherapy procedures (KT group, mean age 42 ± 4.2 years). There was no significant difference in mean age or body mass index (BMI) between the studied groups. Both groups consisted of healthy volunteers who qualified for the studies to promote and increase wellness only and were not previously exposed to WBC. 

Before the research, each subject had a resting electrocardiogram. Then, all subjects were investigated by a physician. On the basis of the obtained data, possible contraindications disqualifying subjects from such a form of treatment were evaluated to eliminate any contraindications towards WBC and kinesiotherapy as well as any coexisting diseases. Prior to each WBC procedure, systolic and diastolic blood pressures were measured [1]. The studied subjects were not allowed to use either drugs or alcohol, or any immunomodulators, immunostimulators, hormones, vitamins, minerals, or other substances with antioxidant properties for the period of four weeks before and during the research. Additionally, each subject was asked to avoid consuming caffeine 12 h prior to laboratory analyses. During the research, the subjects maintained the same mode of physical activity and their diet was not modified [15,19]. The demographic characteristics of the research subjects are presented in Table 1.

### 2.2. Whole-Body Cryostimulation and Kinesiotherapy Procedures

In this research we used the scheme of WBC and kinesiotherapy procedures developed and presented in our earlier works [8,15,19]. The WBC group was exposed to a cycle of WBC procedures with a subsequent session of kinesiotherapy, while, the KT group was subjected to a session of kinesiotherapy only. WBC procedures were conducted by means of 10 daily entries into a cryochamber. The WBC procedures were performed every day, five days a week, from Monday to Friday, at the same time in the morning. They were performed in a Wroclawski-type cryochamber (Creator, Poland). The refrigerant cooling the cryochamber was liquid nitrogen. The cryochamber was built in two parts—an antechamber with the temperature −60 °C and a proper chamber, where the temperature reached −120 °C. The cryochamber was covered with wood and had doors that the subjects could easily open at any time to interrupt a procedure if a temporary indisposition occurred. The subjects entered the chamber in groups of four. The subjects accompanied by a therapist dressed up in the cold-protecting wear entered the antechamber. Here, the therapist once more instructed the subjects on how to behave during the WBC procedure. Then, through linking doors subjects entered the proper chamber one by one without any assistance. Each WBC treatment lasted 3 min at −120 °C. Before entry to the cryochamber each subject stayed for 30 s in the antechamber to adapt to cryogenic temperatures. After an adaptation period in the antechamber, the subjects entered the proper chamber, where they slowly moved in a circle, one after the other. Cryogenic temperatures applied during a procedure in the proper chamber require protection against injury of the parts of the body most exposed to low temperatures. During the WBC procedure, the subjects wore only shorts, socks, wooden clogs, gloves, and ear protectors to protect against frostbite, and their noses and mouths were secured with a surgical mask. Such dress allowed for contact of a greater body area with cold air in order to get better results from the treatment. Glasses, contact lenses, and all jewelry were removed before entering the chamber. Before the WBC procedure the subjects’ bodies were also thoroughly dried to eliminate sweat droplets that could lead to frostbite. All the subjects were informed about shallow breathing. Inhaling should be two times shorter than exhaling due to decompression of cooled air in lungs. Non-compliance with this recommendation may lead to serious breathing depression. During the entire procedure, subjects were in eye contact, through a spyhole, with the physician and staff. When the WBC procedure was finished, subjects left the proper chamber and went back to the antechamber. Then, after the doors were closed, they left the antechamber one by one [8,15,24].

Directly after leaving the cryogenic chamber and changing cloths to a tracksuit, the subjects underwent kinesiotherapy lasting for one hour. The program of the kinesiotherapy procedure was the same for all the subjects in both studied groups. It consisted of exercises on stationary bikes lasting 20 min (5 min,3 rate of perceived exertion (RPE); 4 min, 5 RPE; 2 min, 7 RPE; 4 min, 5 RPE; and 5 min, 3 RPE), treadmill (20 min walking at 4.0 mph), and whole-body stretching exercises lasting 20 min. All the exercises were carried out under the supervision of physiotherapists [8,15,19]. All the subjects completed the studies. WBC procedures were well tolerated by the subjects, no significant complications nor side effects were observed in any of them after applied therapy.

### 2.3. Blood Sample Collection

Blood samples were collected two times—before the beginning of the experiment and after its completion. Fasting blood samples were taken from the basilic vein, using the S-Monvette (Sarstedt) system, into tubes with ethylenediaminetetraacetic acid tri-potassium salt (1.6mg EDTA-K3/mL blood, 2,7mL) and tubes with a clot activator (2.7 mL blood). After centrifugation of samples for 10 min, 900 g of 4 °C obtained serum and EDTA-plasma were immediately aliquoted and stored at a temperature of –75 °C, for future biochemical analyses. Remaining erythrocytes in EDTA tubes were rinsed three times with 0.9% sodium chloride solution buffered with 10mM phosphate (pH 7.4) and, after final centrifugation, lysed in 10mM TRIS/HCl buffer (pH 7.4). Hemoglobin concentration in hemolysates was assayed with Drabkin’s reagent. Repeatability and reproducibility of cyanmethemoglobin method were 1.3% and 2.5% respectively.

### 2.4. Biochemical Analyses

#### 2.4.1. Inflammatory and Endothelium Function Parameters

Myeloperoxidase (MPO) activity in serum was determined with use of a Sigma-Aldrich Myeloperoxidase Colorimetric Activity Assay Kit (Cat. No MAK068). In this method, MPO catalyzes the formation of hypochlorous acid, which reacts with taurine to form taurine chloramine. Taurine chloramine reacts with the chromophore TNB (thionitrobenzoic acid), resulting in the formation of the colorless product DTNB (dithionitrobenzoic acid). One unit of MPO activity is defined as the amount of enzyme that hydrolyzes the substrate and generates taurine chloramine to consume 1.0 µmole of TNB per minute at 25 °C. MPO activity is reported as mU/mL serum (nmole/min/mL). Coefficients of variation for intra- and inter-assay were 6.4% and 8.2%, respectively.

Serum amyloid A concentration in serum was assayed with use of a RayBio^®^ Human SAA ELI-SA kit (RayBiotech Inc., Cat. No ELH-SAA-001). Serum samples were diluted 500-times in Assay diluent C and analysis was performed according manufacturer’s instruction. SAA results are reported as µg/mL. Coefficients of variation for intra- and inter-assay were 3.7% and 5.6%, respectively.

Soluble CD40 Ligand (sCD40L) in serum was assayed by ELISA methods with a DRG Instruments GmbH (Germany) kit (Cat. No EIA-4851/sCD40 Ligand human ELISA). The sCD40L concentration was expressed in ng/mL. The inter- and intra-assay coefficients of variations (CV) were 3.8% and 7.1%.

#### 2.4.2. Oxidative Stress Parameters

Paraoxonase-1 (PON-1) activity was assayed in serum samples using the spectrophotometric kinetic method in which paraoxon (o,o-diethyl-o-(***p***-nitrophenyl)-phosphate; Sigma-Aldrich, Cat. No. D9286, Sigma-Aldrich, MO, USA) was used as a substrate [25]. Ten minutes before analysis, for inactivation of serum cholinesterase, physostigmine salicylate was added to samples to final concentration of 5 mmol/l. Assays were performed on a TECHNICON RA-XT (Technicon Instruments Corporation, Tarrytown, NY, USA) biochemical analyzer at 37 °C and 405 nm. It was assumed that one international unit (1 IU) of paraoxonase-1 is the amount of enzyme that hydrolyzed 1 micromole of paraoxon per minute under assay conditions (0.1 M TRIS/HCl buffer, pH 8.0 and 2 mM calcium ions). For enzyme activity calculation the molar extinction coefficient (E405 = 18290 M−1 cm^−1^) was assumed and the results in serum samples were expressed as IU/l. Repeatability and reproducibility of PON-1 method were 2.7% and 4.5%, respectively.

The ferric-reducing ability of plasma (FRAP) was used as total antioxidants assay and performed in EDTA plasma samples according to Benzie and Strain [26]. The principle of method is the reduction by antioxidant substances presented in plasma of ferric ions. Next, arising ferrous ions react with 2,4, 6-Tri(2-pyridyl)-s-triazine (TPTZ) producing a violet complex. Assays were done on a Flexor/Selectra E biochemical analyzer at 37 °C as end-point colorimetric method. For calibration, synthetic vitamin E analog, Trolox ((±)-6-Hydroxy-2,5,7,8-tetramethylchromane-2-carboxylic acid; Sigma-Aldrich, Cat.no. 238813 USA), was used and antioxidant equivalent concentrations in plasma were expressed as mmol/L. The inter- and intra-assay coefficients of variations (CV) were 1.1% and 3.8%, respectively.

The total oxidant capacity (TOS) in serum samples was determined by application of Erel’s method [27]. Reaction between hydroperoxides present in the serum sample and the reagent (ferrous ions, o-dianisidine and xylenol orange in acidic environment) produced color, with intensity being determined spectrophotometrically. Assays were done on a Flexor/Selectra E biochemical analyzer at 37 °C. For calibration, aqueous solutions of hydrogen peroxide, were used. Results are presented in µmol/l, as hydrogen peroxide equivalents. Repeatability and reproducibility of the TOS method were 2.2% and 6.3%, respectively.

#### 2.4.3. Assay of Intima-Media Thickness

The measurement of intima-media thickness (IMT) was performed in both right and left common carotid arteries. The examination was performed by a vascular medicine specialist who was unaware of the subject’s clinical state. During the examination, subjects lay on their backs with their heads facing backwards and away from the examination side. An average of two measurements was calculated. Measurements were made using a Logic-5 ultrasound system with a 11–15 MHz vascular transducer. The IMT was presented in mm [28].

### 2.5. Statistical Analyses

For statistical analysis, the statistical package of Statistica 10 Pl software was used. For each parameter, the indicators of descriptive statistics were determined (mean value and standard deviation, SD). The normality of the data distribution was checked using the Shapiro–Wilk test, while the homogeneity of the variance was checked by applying the Levene’s test. In order to compare differences between groups, either an independent sample Student t test was used or, alternatively, the Mann–Whitney U test. In the case of dependent samples, the Student t test was used or alternatively the Wilcoxon test. Differences at the significance level of *p* < 0.05 were considered as statistically significant.

## 3. Results

### 3.1. Inflammatory Endothelium Parameters 

The WBC group of subjects after the completion of a cycle of cryostimulation procedures with subsequent kinesiotherapy, had a significant decrease in serum sCD40L level. The serum sCD40L level also showed a decreasing tendency in the KT group. In addition, the difference of serum sCD40L level prior to post-treatment values (Δ) in the WBC group was significantly higher in comparison to the KT group.

Serum MPO activity decreased significantly in the WBC group of patients who underwent a ten-da- long cycle of WBC procedures with subsequent kinesiotherapy after completion of the treatment. In contrast, in the KT group who underwent only kinesiotherapy procedures no significant changes of this parameter were observed after the treatment. In addition, the difference of serum MPO activity prior- to post-treatment values (Δ) in the WBC group was significantly higher in comparison to the KT group. In turn, serum SAA levels decreased significantly in both studied groups after completion the treatment (Table 2).

### 3.2. Oxidative Stress Parameters

Serum PON-1 activity decreased significantly after the completion the treatment in both research groups, but was higher after completion of the treatment in the KT group of subjects who underwent a cycle of kinesiotherapy only than in the WBC group of subjects who underwent a ten-day cycle of WBC procedures with subsequent kinesiotherapy. 

The subjects in the WBC group had, after the completion of the treatment, a statistically-significant increase in the plasma activity of FRAP. After completion of the treatment, FRAP values were significantly higher in the WBC group when compared to the KT group. In the KT group of subjects who underwent a cycle of only kinesiotherapy, without previous cryostimulation procedures, FRAP activity did not change significantly after the treatment, in comparison to the initial values before the beginning of the kinesiotherapy cycle. However, the difference of plasma-FRAP activity prior to post-treatment values (Δ) in the WBC group was significantly lower in comparison to the KT group.

In the WBC group of subjects who underwent a ten-day cycle of WBC procedures with subsequent kinesiotherapy, serum TOS level decreased significantly after the treatment. The difference of serum TOS level prior to post-treatment (Δ) in the WBC group was significantly higher in comparison to the KT group. In the KT group no significant change in the level of serum TOS was observed after the treatment (Table 3).

## 4. Discussion

A growing global rivalry in the present world has led to much of humanity working both longer hours and more intensely to reach even a reasonable level of success. Many of us have lengthened our work-time at the cost of proper rest and sufficient sleep. A lack of regular sleep, an excess of stress, work overload, and the increasing tempo of everyday life causes deterioration in the condition of human body and favors the development of disease. For these reasons we seek more effective methods of well-being, which will allow for an intensification of the physiological processes for well-being and contribute to the improvement of physical efficiency, mood, and psychological state. A method of physical medicine that fulfills the previously-mentioned conditions and is therefore becoming more frequently-applied in well-being is whole-body cryostimulation (WBC).

The effect of WBC treatment may depend on the type of cryochamber in which procedures are performed, the temperature and number of procedures, and whether WBC procedures are connected with a subsequent session of kinesiotherapy [19]. This is why, in this research, we used the scheme of WBC and kinesiotherapy procedures developed and presented in our earlier works [8,15,19,28] to be able to compare the obtained results with the other ones in the same circumstances. 

In our study we observed that, after the completion of treatment, the WBC group of healthy subjects who underwent a ten-day cycle of WBC procedures with subsequent kinesiotherapy had a significant decrease in serum activities of PON-1 and MPO as well as of serum TOS and sCD40L or SAA levels. In addition, after the completion of treatment the WBC group of subjects had a significant increase in plasma-FRAP values.

The research presented is the first to estimate the impact of WBC procedures with subsequent kinesiotherapy on activities of PON-1 and MPO as well as on sCD40L and SAA levels in healthy subjects. 

The observed changes of the parameters studied might be connected with reduction in oxidative stress, lipid profile parameters, or the anti-inflammatory action of WBC procedures [7,8,18,19].

Very few papers have estimated the impact of WBC procedures on the prooxidant–antioxidant balance in healthy subjects. They have proved that WBC procedures could lower oxidative stress. In the study by Duque et al. [29], a significant rise was seen in the total antioxidant status (TAS) value in healthy male subjects at the end of a cycle of 45 procedures of WBC performed three times a week. In addition, Miller et al. [30] found that 10 sessions of WBC may decrease the production of different reactive oxygen and nitrogen species in healthy subjects caused by a rise in the plasma levels of, uric acid (UA) and TAS as well as superoxide dismutase (SOD) activity. These findings are similar to our previous papers [15,19]. In contrast, Lubkowska et al. [31] presented that a single session of WBC could induce disturbances in prooxidant–antioxidant balance caused by decreasing TOS and temporarily, TAS.

WBC treatment may also have a beneficial impact on lipid parameters. In the study [18] the authors observed a drop in total cholesterol, LDL-cholesterol, and triacylglycerols levels, and an increasing HDL-cholesterol level after 20 sessions of WBC in healthy male subjects, but after 10 sessions of WBC, only LDL cholesterol level was lowered, while a simultaneous HDL-cholesterol level rise was noted. In our previous study, we observed a significant drop in total cholesterol, LDL-cholesterol, and triacylglycerol levels after a ten-day cycle of WBC procedures with subsequent kinesiotherapy in healthy male subjects. In current study, the HDL-Ch level did not change significantly in the WBC group after the completion of the treatment [19]. Cryogenic temperatures applied for the whole human body may also lower ox-LDL level [15].

In a few papers it has been presented that in athletes and healthy subjects WBC procedures lower inflammatory parameters. Pournot et al. [32] noticed that WBC procedures decreased, along with an increase in IL-1β, CRP, and IL-1ra levels after intense exercise. But the levels of TNF-α, IL-10, and IL-6 did not change. In another paper Banfi et al. [17] presented that WBC procedures performed once daily for 5 days caused a rise of anti-inflammatory cytokine (IL-10) and a drop of proinflammatory cytokines (Il-2 and IL-8) in athletes. Moreover, the beneficial impact of WBC procedures on inflammatory parameters was also observed in patients with rheumatoid arthritis [33] and ankylosing spondylitis [7,34].

In our research, the observed drop in PON1 activity, after the completion of WBC treatment, may be connected with the reduction of the accumulation of oxidized lipids on the HDL particle, or the inhibition the oxidation of lipid on LDL [35,36]. The decrease in ox-LDL level, after WBC procedures, was observed in our previous studies [15]. The observed effect of PON-1 fall may be also associated with the decrease of homocysteine thiolactone production. It has been shown that this molecule is the physiological substrate for paraoxonases (thiolactonase activity of PON-1) and it probably plays the regulatory function on its activity [37]. Decreasing of TOS can also affect PON-1 activity, because lower availability of lipid (hydro)peroxides (assayed in the presented work as TOS) requires lower activity of the enzyme that degrade them.

Also, after the WBC procedures, the observed decrease in myeloperoxidase activity may have a relationship with the reduction of inflammation and white blood cell activation, as well as LDL oxidation and nitric oxide consumption leading to endothelial dysfunction. Increased activity of myeloperoxidase can be a key factor in oxidative modification of LDL particles in subendothelial space and local generation of oxidative and nitrosative stress [38,39].

In our study we also observed that, after the completion of treatment, the WBC group of healthy subjects who underwent a ten-day cycle of WBC procedures with subsequent kinesiotherapy had a significant decrease in serum SAA levels. SAA is an acute-phase protein, which is associated with high-density lipoprotein (HDL) particles. Recent studies have shown it may also play a role in the pathogenesis of chronic inflammatory diseases, such as rheumatoid arthritis and atherosclerosis [40]. Thus, changes in PON-1 activity and SAA serum concentration may be associated with an alternation of HDL composition and its antiatherogenic properties.

In addition, we observed a significant decrease in serum CD40L in the healthy WBC group subjects after the completion of treatment. CD40 is a proinflammatory signaling protein, which is among others, expressed on vascular endothelium. Ligation of CD40 on endothelial cells leads to production of proinflammatory cytokines and an enhanced expression of CD54 (intracellular adhesion molecule-1 (ICAM-1)), CD62E (E-selectin), and CD106 (vascular cell adhesion molecule-1 (VCAM-1)) with a consequent increase in leucocyte binding [41]. The confirmation of this fact may be in the papers reporting that WBC procedures may decrease the levels of ICAM-1 [15,17].

To sum up, in healthy subjects after the completion of WBC treatment, the observed decrease in serum activities of PON-1 and MPO as well as serum TOS and sCD40L or SAA levels and the increase in plasma-FRAP values indicates that WBC procedures have a beneficial impact on human endothelial cells homeostasis. The WBC procedure mechanisms are still not fully known and more studies are needed in the same circumstances. It will be interesting to see studies that tie the WBC procedures and current biomarkers presented in this work to the inflammatory changes in lipid profiles reported in other works.

## 5. Conclusions

Whole-body cryostimulation procedures followed by kinesiotherapy have a beneficial impact on inflammatory endothelium parameters in healthy subjects, therefore the treatment may be useful as a wellness method in healthy subjects. However, further research, including clinical trials, is definitely needed.

## Figures and Tables

**Table 1:** Demographic characteristics of the research subjects.

Characteristic	WBC Group (*n* = 32)	Kinesiotherapy Group (*n* = 16)	*p* Value
Age, years, mean (SD)	43 ± 3.9	42 ± 4.2	0.673
Sex M/F	16/0	16/0	-
BMI, kg/m^2^, mean (SD)	24.83 ± 4.7	23.26 ± 6.3	0.858
Smoking (yes/no)	0/16	0/16	-
Carotid IMT (mm)	0.57 ± 0.11	0.55 ± 0.06	0.83

WBC: whole-body cryostimulation; SD: standard deviation; BMI: body mass index; IMT-intima-media thickness.

**Table 2:** Endothelium and inflammatory parameters (mean value ± standard deviation (SD)) in healthy subjects before and after the completion of a cycle of ten whole-body cryostimulation procedures with subsequent kinesiotherapy (WBC group) or a cycle of ten kinesiotherapy-only procedures (KT group), with statistical analyses. p, plasma; s, serum; e, erythrocyte lysates; Δ, difference prior to post-treatment.

Parameters		WBC Group	KT Group	*p*
sCD40L(s) [ng/mL]	Before	6.63 ± 1.01	3.70 ± 2.06	**0.0006**
	After	4.84 ± 1.10	2.72 ± 1.83	**0.0005**
	*p* *	**<0.0001**	0.075	
	**Δ**	−1.79 ± 0.98	−0.98 ± 2.05	**0.03**
MPO (s) [mU/mL]	Before	246.80 ± 117.39	206.34 ± 41.96	**0.935**
	After	152.39 ± 63.14	257.11 ± 145.01	<0.001
	*p* *	**0.008**	0.109	
	Δ	−94.41 ± 131.09	60.37 ± 133.99	**0.005**
SAA (s) [µg/mL]	Before	4.15 ± 1.88	3.94 ± 1.61	0.91
	After	0.97 ± 1.52	0.66 ± 1.08	0.55
	*p* *	**0.001**	**0.0008**	
	**Δ**	−3.17 ± 2.6	−3.29 ± 2.11	0.88

*p*, statistical significance of differences between both groups of subjects; *p* *, statistical significance of differences between values before and after treatment in particular groups of subjects.

**Table 3:** Oxidative stress parameters (mean value ± standard deviation (SD)) in healthy subjects before and after the completion of a cycle of ten whole-body cryostimulation procedures with subsequent kinesiotherapy (WBC group) or a cycle of ten kinesiotherapy procedures only (KT group), with statistical analyses. p, plasma; s, serum; e, erythrocyte lysates; Δ, difference prior to post-treatment.

Parameters		WBC Group	KT Group	*p*
PON-1 (s) [IU/L]	Before	158.2 ± 106.6	152.4 ± 66.7	0.855
	After	129.2 ± 113.1	138.4 ± 55.3	0.077
	*p* *	**0.004**	**0.034**	
	Δ	−29.0 ± 29.6	−14.0 ± 22.6	0.199
FRAP (p) [µmol/l]	Before	643.53.1 ± 116.29	638.44 ± 82.74	0.901
	After	787.56 ± 303.44	608.84 ± 91.40	**0.027**
	*p* *	**<0.001**	0.089	
	**Δ**	14.03 ± 304.60	−29.6 ± 77.8	**0.031**
TOS (s) [µmol/l]	Before	19.68 ± 5.91	10.12 ± 3.28	**<0.001**
	After	15.21 ± 4.85	11.97 ± 6.71	0.06
	*p* *	**0.005**	0.485	
	**Δ**	−4.47 ± 7.46	1.85 ± 7.80	**0.009**

*p*, statistical significance of differences between both groups of subjects; *p* *, statistical significance of differences between values before and after treatment in particular groups of subjects.
